# Metabolism and iron signaling in ferroptotic cell death

**DOI:** 10.18632/oncotarget.5671

**Published:** 2015-09-15

**Authors:** Minghui Gao, Prashant Monian, Xuejun Jiang

**Affiliations:** Cell Biology Program, Memorial Sloan-Kettering Cancer Center, New York, NY, USA

**Keywords:** ferroptosis, transferrin, L-Glutamine, metabolism, iron

Programmed cell death (PCD) plays critical roles in development and tissue homeostasis of multicellular organisms, and malfunction of programmed cell death contributes to the pathogenesis of a variety of human diseases, including cancer [1]. While apoptosis had been the main focus of the PCD study for decades, recent advances demonstrate that there exist multiple other forms of cell death that are also genetically “programmed”, such as receptor-interacting protein kinase 3 (RIPK3)-dependent necroptosis and iron-dependent ferroptosis [1].

Ferroptosis was initially described by Stockwell and colleagues as a unique type of cell death induced by a synthetic compound, erastin [2]. Mechanistically, ferroptosis is distinct from apoptosis and RIP3-dependent necrosis, and is dependent on cellular iron (thus the name) and reactive oxygen species (ROS). Erastin functions to inhibit System XC^−^ cystine/glutamate antiporter, thus abrogating cystine import and downstream glutathione synthesis. This leads to deregulated cellular redox homeostasis and ultimately, ferroptotic cell death. Interestingly, it was reported previously that an excess of glutamate can prevent System XC^−^ cystine/glutamate antiporter from importing cystine and subsequently induce a type of cell death called oxytosis or glutamate-induced oxicytotoxicity [1, 3]. Hence oxytosis and ferroptosis most likely share the same central mechanisms. Notably, although inhibition of System XC^−^ antiporter or elimination of its subunit SLC7A11 triggers massive ferroptosis in cultured cells, SLC7A11-knockout mice are viable and fertile [4]. A possible reason for this striking difference is that in cell culture experiments, cysteine in culture media is rapidly oxidated to cystine, thus cells solely rely on cystine imported by System XC^−^ antiporter to counter ferroptosis. On the other hand, *in vivo* plasma cysteine might be sufficient to maintain cellular redox homeostasis.

Ferroptosis has been implicated in diseases such as ischemia-induced organ damage [5]. Importantly, it was demonstrated most recently that ferroptosis also contributes to the tumor suppressive function of p53; loss of p53 leads to transcriptional upregulation of SLC7A11, thus rendering cancer cells more resistant to ferroptotic cell death [6]. Although ferroptosis is strongly implicated in human diseases, the precise molecular mechanisms and biological functions of ferroptosis remain poorly understood.

Our study of ferroptosis started from an intriguing and rather unexpected observation. Nutrient status is a crucial parameter for the cell to make life-and-death decisions. In our efforts to understand how nutrient signaling impacts cell death, we observed, to our surprise, that upon amino acid starvation, the presence of serum promoted, rather than mitigated, cell demise [7]. Such serum-induced cell death is distinct from apoptosis and necroptosis, and shows clear morphological features of necrosis [7]. Classical biochemical fractionation coupled with mass spectrometry analysis pinned down transferrin and L-glutamine as the serum factors necessary and sufficient to induce potent necrosis upon amino acid starvation [7]. The identities of these killing factors came as a big surprise, since both transferrin and L-glutamine are normally required for cell viability! Yet in the PCD field, seemingly counter-intuitive findings are the norm, as even the life-essential protein cytochrome C can be a murderer given the “right” conditions. Because transferrin is an iron carrier protein and as we further found that in the presence of serum (or transferrin plus L-glutamine), cysteine/cystine-deprivation alone can trigger this type of cell death as potently as pan-amino acid starvation, we tested if we were studying ferroptosis, which also requires iron and can be triggered by cystine starvation. Comprehensive tests confirmed that indeed we were, and indeed ferroptosis requires the extracellular factors transferrin and L-glutamine [7]. Thus, a bit disappointedly, we did not get a chance to coin a new name for this type of cell death. However, within our lab we do prefer our unofficial term “metaptosis” over “ferroptosis” (“meta” for both the metal iron and the cellular metabolic process glutaminolysis). Ultimately, confirmation of the *in vivo* role of iron is needed to justify these new terms.

After the initial surprise and drama, the players and mechanisms downstream of transferrin and L-glutamine all fell into place in a rather logical and straightforward fashion. Transferrin receptor is required for ferroptosis [7], most likely through mediating iron-dependent transferrin transportation. The glutamine transporters SLC1A5/SLC38A1 and the metabolic process glutaminolysis are both essential for ferroptosis, as demonstrated by pharmacological and RNAi knockdown experiments [7]. We further showed using an ex vivo experiment that inhibition of glutaminolysis, an essential component of ferroptosis, can reduce heart injury trigged by ischemia/reperfusion stress.

Taken together, we discovered multiple novel molecular players of ferroptosis and its intimate interplay with cellular metabolism and redox machinery, and revealed molecular targets for related disease intervention. Many important questions remain for further investigation. To name a few, mechanistically, what is the molecular executioner of ferroptosis (as caspases are for apoptosis), and how do glutaminolysis and iron signaling communicate with this molecule? Biologically, does ferroptosis play any beneficial function during normal development, and if so, what developmental cue triggers ferroptosis? This question is particularly important in the PCD field, because, in order to qualify the term PCD, a death process not only needs to be dictated by a genetically encoded molecular pathway, its occurrence should also exert certain biologically beneficial function(s). Another exciting area that should be explored is the potential role of ferroptosis in disease pathogenesis and treatment. While the role of ferroptosis in ischemia and related degenerative diseases is relatively clear, the role of ferroptosis in cancer appears to be more complicated. On one hand, the tumor suppressor p53 can promote ferroptosis. But on the other hand, glutaminolysis, a metabolic process often upregulated in cancer cells and required for cancer cell viability, is also required for ferroptosis. Therefore, if a ferroptosis-promoting cancer therapy were developed, would certain cancers be more sensitive to such therapy because of their glutaminolysis-dependence (an Achilles heel)? We foresee that the study of ferroptosis will thrive in the coming years.

## References

[1] Vanden Berghe T. (2014). Nat Rev Mol Cell Biol.

[2] Dixon S. J. (2012). Cell.

[3] Murphy T. H. (1989). Neuron.

[4] Sato H. (2005). J Biol Chem.

[5] Friedmann Angeli J. P. (2014). Nat Cell Biol.

[6] Jiang L. (2015). Nature.

[7] Gao M. (2015). Mol Cell.

